# Dangerousness Index in Forensic Psychiatric Examination: A Tool for Aiding Medical Decision Regarding the Risk of Antisocial Acts

**DOI:** 10.3390/diagnostics15081004

**Published:** 2025-04-15

**Authors:** Daniela Margareta Varga, Florica Voiță-Mekeres, Gabriel Mihai Mekeres, Călin David Buzlea, Lavinia Davidescu, Camelia Liana Buhas

**Affiliations:** 1 Doctoral School of Biomedical Sciences, University of Oradea, 410073 Oradea, Romania; varga.danielamargareta@student.ro (D.M.V.); cameliabuhas@uoradea.ro (C.L.B.); 2 County Clinical Emergency Hospital of Oradea, 410087 Oradea, Romania; 3 Department of Morphological Disciplines, Faculty of Medicine and Pharmacy, University of Oradea, 410087 Oradea, Romania; 4 Medical Department, Faculty of Medicine and Pharmacy, University of Oradea, Universitatii Street Nr. 1, 410087 Oradea, Romania; lavinia.davidescu@didactic.uoradea.ro; 5 Surgical Department, Faculty of Medicine and Pharmacy, University of Oradea, Universitatii Street Nr. 1, 410087 Oradea, Romania; buzlea.calindavid@didactic.uoradea.ro

**Keywords:** dangerousness index, forensic psychiatric examination, new tool, validation

## Abstract

**Background and Objectives**: The assessment of dangerousness and risk of recidivism are crucial aspects of forensic psychiatric evaluations, influencing therapeutic and security measures. This study aimed to develop and validate a new tool, the Dangerousness Index in Forensic Psychiatry (IPPML), following a psychometric scale construction methodology. **Materials and Methods:** The sample consisted of 261 participants (157 males, 104 females) aged 19–75 years, divided into an experimental group (*n* = 126) with a history of forensic psychiatric examination and a control group (*n =* 135) diagnosed with schizophrenia. **Results**: Exploratory factor analysis revealed two factors, Performance and Social, explaining 45.55% of the data variance. The IPPML demonstrated adequate internal consistency (α = 0.881) for the entire sample, with Factor 1 showing strong consistency (α = 0.896) and Factor 2 exhibiting acceptable consistency (α = 0.628). Reliability ranged from 89.6% to 62.8% when administered to participants with psychoses undergoing forensic psychiatric evaluation, decreasing to 42.5% for legally evaluated patients and increasing from 58.7% to 84.3% for participants with schizophrenia without forensic psychiatric evaluation. Discriminant validity analysis indicated higher psychiatric dangerousness with forensic implications in males. **Conclusions**: The IPPML shows promise as a tool for assessing dangerousness in forensic psychiatry and aiding medical decision-making regarding the risk of antisocial and potentially harmful acts.

## 1. Introduction

The assessment of dangerousness and risk of recidivism is critical in forensic psychiatric evaluations, impacting therapeutic and security measures.

Forensic-psychiatric risk assessment of persons in prisons, deemed dangerous due to mental disorder and crime, is ubiquitous [1]. Structured instruments are often used to examine risk and make risk management plans [2], but have limitations [2,3,4]. Forensic assessment informs judicial decisions on incarceration, treatment, privileges, and release from prisons [5,6]. The forensic mental health system balances risk management to avoid future harm to others and provides treatment to incarcerated persons with severe mental illness [3,7].

Accurately assessing potential dangerous behavior in forensic psychiatric patients is essential for public safety and effective treatment planning.

Risk assessment and management of dangerous behavior in forensic psychiatric patients are crucial aspects of forensic mental health services. The Historical, Clinical and Risk Management Scales (HCR-20) effectively predict violent and non-violent offending following release from medium-secure units [8]. The HCR-20 total score demonstrated good predictive validity, particularly for short-term periods up to one year post-discharge. While risk assessment tools are widely used, their effectiveness in preventing recidivism remains uncertain. A cluster randomized controlled trial found no significant difference in violent or criminal incidents between patients receiving risk assessment with the Short Term Assessment of Risk and Treatability (START) combined with shared care planning, and those receiving usual care [9]. This highlights the complexity of translating risk assessment into effective interventions. Forensic mental health services must balance public protection with ethical patient care [10], considering factors beyond acute symptomatology in risk assessment. Ongoing research is needed to improve risk assessment and management strategies in forensic psychiatric settings. Current tools for evaluating dangerousness in forensic psychiatry have limitations, including varying degrees of reliability and validity. Several studies have examined various risk assessment instruments with mixed results. The V-RISK-10, a brief 10-item checklist, demonstrated good predictive validity for inpatient violence, with an area under the curve (AUC) of 0.83 and sensitivity/specificity of 0.81/0.73 [11]. It also showed validity for predicting violence after discharge from acute psychiatric wards, with AUC values of 0.80 and 0.75 for 3 and 12-month follow-ups, respectively [12]. However, other tools have shown more modest results. A study on female forensic psychiatric patients found overall moderate predictive validity for recidivism but low validity for violence specifically across several instruments including the HCR-20, START, and PCL-R [13]. Some research suggests incorporating neuroimaging data may improve predictive performance. A study found including resting-state cerebral blood flow measurements increased the AUC from 0.69 to 0.81 for predicting recidivism in forensic psychiatric patients [14]. However, this approach faces ethical challenges and requires further validation. While some promising tools exist, there remains a need for more reliable and valid instruments for assessing dangerousness in forensic psychiatry. Further research is needed to refine existing tools and develop new approaches that can more accurately and consistently evaluate risk across diverse forensic populations.

There is a critical need for a psychometrically sound tool that reliably assesses dangerousness and risk of recidivism in forensic psychiatric populations. Developing a robust instrument like the Dangerousness Index in Forensic Psychiatry (IPPML) is essential to enhance the accuracy of forensic psychiatric evaluations and to inform better medical and legal decisions. This study aims to develop and validate the IPPML using a rigorous psychometric scale construction methodology. The IPPML scale shows high internal consistency, and significant differences in dangerousness scores are observed between male and female participants. Furthermore, we expect that the instrument will demonstrate high reliability and strong discriminant validity—evidenced by higher scores among individuals undergoing forensic psychiatric evaluation compared to those diagnosed solely with schizophrenia.

## 2. Materials and Methods

### 2.1. Participants

The study included 261 participants (M = 47.76, SD = 12.81), consisting of 157 males (60.2%) and 104 females (39.8%). Among them, 122 (46.7%) were from rural areas, while 139 (53.3%) resided in urban areas. The experimental group comprised 126 participants (M = 48.53, SD = 12.73), including 76 males (60.3%) and 50 females (39.7%). The control group consisted of 135 participants (M = 47.04, SD = 12.89), with 81 males (60%) and 54 females (40%).

### 2.2. Data Collection

Participants were recruited from Spitalul de Psihiatrie și pentru Măsuri de Siguranță Ștei. The experimental group consisted of individuals with a documented history of forensic psychiatric evaluation at the institution, who were identified through case file reviews and clinician referrals during scheduled assessments or follow-up visits. In parallel, the control group, comprising individuals diagnosed with schizophrenia, was recruited from both inpatient and outpatient services within the same institution. All potential participants were screened against predefined inclusion criteria (age ≥ 18 years and having undergone at least one forensic psychiatric evaluation for the experimental group) and exclusion criteria (uncontrolled mental illness or inability to provide informed consent). Written informed consent was obtained from all participants prior to their enrollment, ensuring adherence to ethical standards and enhancing the representativeness of the sample.

### 2.3. Data Analysis

In the first part of the research, we analyze descriptive statistical indicators such as age and gender in relation to the included groups (experimental and control).

We assess the frequencies of statistical indicators regarding hereditary, pathological, and social history based on the type of group (experimental vs. control).

The χ2 test was used to evaluate clinical data measured using ordinal scales, given that the statistical data for each variable appears in the form of frequencies.

The experimental group was analyzed in terms of criminal acts committed, whereas the control group did not present criminal acts or behaviors contrary to societal norms.

We analyzed the following variables:

- Hereditary history based on the type of group.
- Personal pathological history according to participants’ diagnoses.
- Discernment at the time of committing the criminal act.
- Safety measures categorized under Articles 109 and 110.
- Number of hospitalizations.

Development and Validation of the Dangerousness Index in Forensic Psychiatry (IPPML)

To develop a measurement scale for dangerousness in forensic psychiatry, we followed methodological recommendations for psychometric scale construction (Gerbing & Anderson, 1988) [15] as well as the analysis of previous relevant studies in the field (Nenkov, Inman & Hulland, 2008) [16]. The initial item generation was followed by five stages, including (1) scale purification and initial validation, (2) exploration of the nomological network, (3) tests for experimental and predictive validity, (4) verification of predictive validity, and (5) reliability assessment. Both qualitative and quantitative data were analyzed to achieve the study’s research objectives.

### 2.4. Item Generation

A broad set of items was developed to cover all potential aspects of dangerousness in forensic psychiatry. A group of 10 experts evaluated each statement for content validity and formal validity. To ensure item relevance for both research and forensic practice, the panel included five university professors from similar institutions and five medical specialists. These experts rated each item on a 5-point scale, ranging from “Strongly Disagree (1)” to “Strongly Agree (5)”.

Experts selected five items that best fit the construct. Items scoring above 3 or selected by at least one expert were retained. This process reduced the item list further. Only items assigned a priori by at least 60% of evaluators were kept. After this process, 20 potential items remained (Table 1).

### 2.5. Inclusion and Exclusion Criteria

The inclusion criteria for the study required participants to have undergone at least one forensic psychiatric examination (FPE), to be 18 years or older at the time of the examination, and to have provided informed consent.

Exclusion criteria included individuals who declined participation, those under the age of 18, and individuals with uncontrolled mental illnesses.

### 2.6. Ethics

All participants voluntarily agreed to take part in the study and provided written informed consent before their inclusion. Ethical approval was granted by the Ethics Committee of Spitalul de Psihiatrie și pentru Măsuri de Siguranță Ștei under reference number 14,493/26.11.2021, in accordance with national and international ethical guidelines for research involving human subjects. The study adhered to the principles outlined in the Declaration of Helsinki and ensured confidentiality and data protection in compliance with relevant regulations.

**Table 1:** IPPML items in the initial research phase.

No.	Item
1	Have you ever been diagnosed with a mental illness?
2	Does anyone in your family have a diagnosed mental illness?
3	Have you ever suffered a traumatic brain injury?
4	Have you consumed psychoactive substances, alcoholic or non-alcoholic, before committing the act?
5	Have you had sleep disturbances in the last two weeks?
6	Have you had problems with the police in the past?
7	In childhood, did you commit antisocial acts or mistreat animals?
8	Have you repeatedly run away from school?
9	In childhood, did you lie or steal repeatedly?
10	When wronged, do you feel the urge to hit?
11	Did you plan the act in advance (premeditate it)?
12	How do you react when someone disagrees with you—angry, verbally or physically aggressive?
13	Before committing the act, did you hear voices or messages (God speaking, messages from TV, etc.)?
14	Can you maintain your job?
15	Do you relate well with colleagues at work/school?
16	Do you understand the gravity of your actions?
17	Can you talk about your problems with friends?
18	Do you regret committing the act?
19	Do you receive support and help from your family?
20	Do you believe you can reintegrate into society?

### 2.7. Statistical Methodology

Preliminary calculations included the mean values and standard deviation (SD) for both participant groups. Statistically significant thresholds were considered below 0.05.

The normality of the data was assessed using the Shapiro–Wilk test. For data with a Gaussian distribution, the mean and standard deviation were reported; for non-Gaussian data, the median and interquartile range (IQR) were provided. Demographic characteristics of participants were analyzed using the Chi-square test or the Freeman–Halton extension of Fisher’s exact test. Examination of the correlation matrix indicated that the IPPML scale items were mostly correlated, and Bartlett’s test of sphericity was statistically significant. The reliability of the IPPML was estimated using Cronbach’s alpha coefficient.

Exploratory factor analysis (EFA) was conducted to examine the composition of potential factors within the IPPML. The Kaiser–Meyer–Olkin (KMO) method was applied to assess sample adequacy. Additionally, Varimax rotation was employed in EFA, allowing factor correlation at different intensities.

Confirming the importance of factors within the IPPML through EFA, as well as validating the scale using statistical methods, will support the usefulness of this tool in forensic psychiatric research and practice. Given that predictors are continuous variables, we employed Multiple Linear Regression for predictive purposes and conducted comparisons between the two experimental groups.

A priori power analysis was conducted using an alpha level of 0.05, which confirmed that our study achieved at least 80% statistical power. A post hoc power analysis shows that our sample size of 261 participants is sufficient to detect the expected effects with reasonable reliability. Using these values in a noncentral t-distribution framework, the estimated statistical power is approximately 98.1%. This suggests that our sample size is more than adequate to detect a medium effect size with high reliability. Rounding up, each group would require approximately 63 participants. Thus, a total sample size of about 126 participants (63 in each group) is needed to ensure 80% power.

All responses were measured on a Likert scale from 1 to 5, with a total score ranging from 20 to 100. All statistical analyses were performed using SPSS version 22 (IBM Corp., Armonk, NY, USA), and the study adhered to the ethical principles outlined in the Declaration of Helsinki.

## 3. Results

A total of 261 participants, aged between 19 and 75 years (M = 47.76, SD = 12.81), were included in the study. By gender distribution, there were 157 males (60.2%) and 104 females (39.8%). Regarding the participants’ residential background, 122 (46.7%) were from rural areas, while 139 (53.3%) were from urban areas.

The experimental group consisted of 126 participants aged between 19 and 75 years (M = 48.53, SD = 12.73), of whom 76 were men (60.3%) and 50 were women (39.7%). The educational level distribution indicated that 36 participants had middle school education (28.6%), 28 had vocational school education (22.2%), 53 had high school education (42.1%), and 9 had higher education (7.1%). Marital status distribution showed that 87 participants were unmarried (69%), 11 were married (8.7%), 21 were divorced (16.7%), 1 was in a cohabiting relationship (0.8%), and 6 were widowed (4.8%). Regarding economic status, 72 participants (57.1%) reported low economic status, 52 (41.3%) medium status, and only 2 (1.6%) high status. Residential background distribution indicated 69 participants (54.8%) were from rural areas and 57 (45.2%) from urban areas.

The control group consisted of 135 participants aged between 20 and 73 years (M = 47.04, SD = 12.89), with 81 men (60%) and 54 women (40%). The educational level distribution indicated that 8 participants had middle school education (5.9%), 29 had vocational school education (21.5%), 79 had high school education (58.9%), and 19 had higher education (14.1%). Marital status distribution showed that 44 participants were unmarried (32.6%), 73 were married (54.1%), 11 were divorced (8.1%), 6 were in a cohabiting relationship (4.4%), and 1 was widowed (0.7%). Regarding economic status, 11 participants (8.1%) reported low economic status, 76 (56.3%) medium status, and 48 (35.6%) high status. Residential background distribution indicated 53 participants (39.3%) were from rural areas and 82 (60.7%) from urban areas (Table 2).

Our objectives were to validate the IPPML scale and establish norms for the population.

We focused on key statistical indicators; therefore, in Table 3, we present the mean and standard deviations for each item based on the type of group. Table 3 shows that the means of the experimental and control groups are relatively close.

### 3.1. Factor Structure

A preliminary data analysis was conducted to capture gender differences in the IPPML. No statistically significant differences (independent t-test based on participants’ gender) were found; therefore, means and standard deviations were calculated for the entire sample.

We consider the IPPML a suitable method for assessing dangerousness in forensic psychiatry and an objective tool for medical decision-making.

A preliminary examination of the correlation matrix demonstrated that the IPPML items are intercorrelated, and Bartlett’s test of sphericity was statistically significant (χ^2^(190, *N =* 261) = 5536.534, *p* < 0.0001), confirming the suitability of exploratory factor analysis (EFA) for adapting the scale to the target population.

The sample adequacy was tested using the Kaiser–Meyer–Olkin (KMO) method, yielding a value of 0.903, confirming that the sample meets basic conditions for conducting EFA. For sample adequacy assessment for each variable, the “anti-image” option (available in SPSS, version 22 (IBM Corp., Armonk, NY, USA)) was used, showing diagonal values greater than 0.90 for 12 items (4, 5, 6, 7, 10, 14, 15, 16, 17, 18, 19, 20), values above 0.60 for three items (8, 9, 11), values above 0.70 for three items (1, 2, 13), and values above 0.30 for one item (3). These values indicate good sample adequacy for most variables.

We applied factor analysis using the Varimax rotation method, which allows factors to correlate at different intensities. Two factors were identified, representing 45.55% of the data variance (Table 4). The identified IPPML factors are statistically relevant and demonstrate the likelihood of two component subscales within the analyzed groups.

After rotation (Table 4), we observe a strong influence of the first factor (9.111), followed by the influence of the second factor (3.883). These results support the credibility of the IPPML instrument in assessing dangerousness in forensic psychiatry.

**Figure 1:**
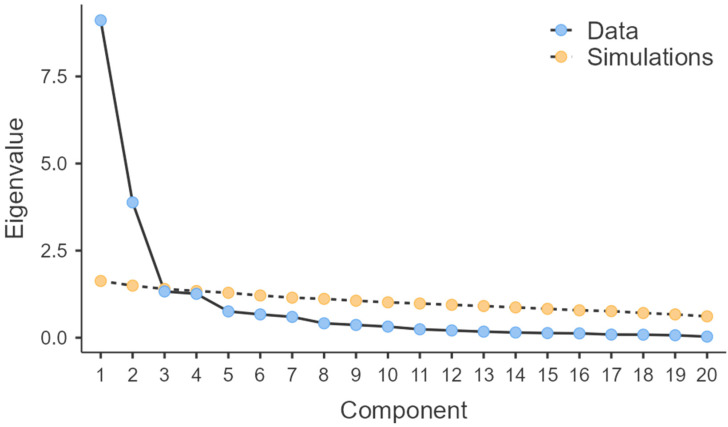
Graphical representation of eigenvalue intensity for each hypothetical IPPML factor.

Figure 1 presents a graphical representation of the eigenvalues for each of the 20 potential factors, plotted on the *x*-axis, while eigenvalues are noted on the *y*-axis. Consequently, we extract two factors (as observed in Table 4) that can lead to an adequate solution in the case of IPPML.

**Figure 2:**
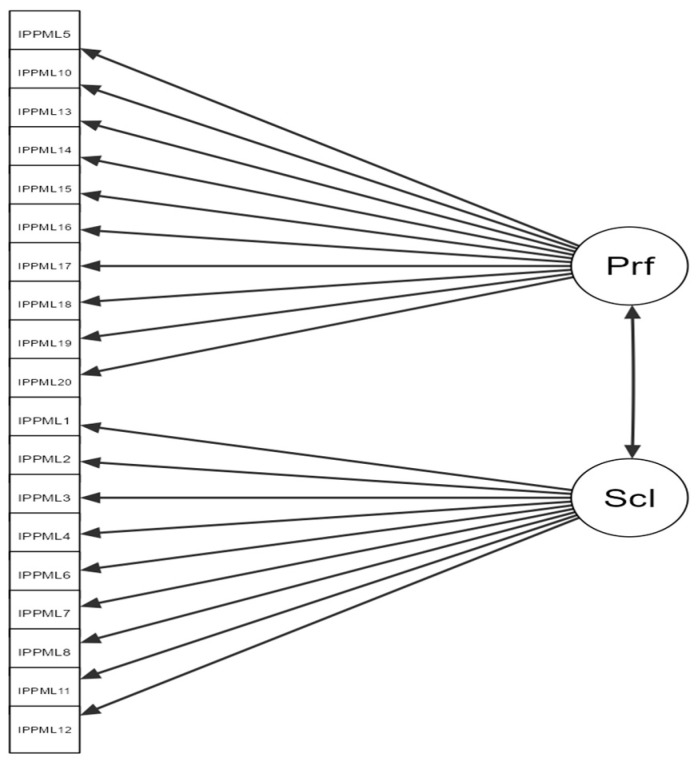
Path diagram of the two-factor (Performance and Social) model for the IPPML.

Table 5 presents the factorial structure matrix, indicating the grouping of items 5, 10, 13, 14, 15, 16, 17, 18, 19, and 20 into the first factor, and items 1, 2, 3, 4, 6, 7, 8, 9, 11, and 12 into the second factor. Table 5 shows how items correlate with the two IPPML factors. Although the scale authors initially considered item 3 (“Have you ever suffered a traumatic brain injury?”) to be part of the presumptive medico-legal investigations, its validity is low, potentially causing difficulties for respondents (Figure 2).

Traumatic brain injuries (TBIs) are associated with various psychiatric and neurobehavioral problems. They cause cognitive, affective, and behavioral sequelae, often more disabling than residual physical effects. Moreover, moderate and severe TBIs can lead to personality changes, including impulsivity, severe irritability, affective instability, and apathy. Even mild TBIs are linked to affective symptoms, suicidality, and exacerbation or onset of multiple psychiatric disorders, including post-traumatic stress disorder (PTSD) and major depressive disorder [17].

Based on these results, we propose a restructuring of the items that may be more aligned with how respondents perceive themselves:

Factor 1—Performance: 10 items (5, 10, 13, 14, 15, 16, 17, 18, 19, 20).

Factor 2—Social: 10 items (1, 2, 3, 4, 6, 7, 8, 9, 11, 12).

### 3.2. Internal Consistency

In our study (Table 6), we found that the total IPPML score presents adequate internal consistency as an indicator of measurement precision (α = 0.881) for the entire sample (*N =* 261). Factor 1 also demonstrates strong internal consistency (α = 0.896), while Factor 2 exhibits acceptable methodological internal consistency (α = 0.628).

In the experimental group, Factor 1 presents low but acceptable consistency (α = 0.622), while for Factor 2 (α = 0.479) and the total IPPML score (α = 0.425), the obtained coefficients indicate reservations regarding their use.

Internal consistency in the control group shows methodologically adequate values for Factor 1 (α = 0.817), acceptable values for Factor 2 (α = 0.587), and high reliability for the total IPPML score (α = 0.843).

The obtained values indicate a reliability range between 89.6% and 62.8% when administered to participants with psychoses undergoing forensic psychiatric evaluation. When explicitly applied to legally evaluated patients, the instrument’s reliability decreases to 42.5%. On the other hand, when used for participants with schizophrenia but without forensic psychiatric evaluation, reliability increases from 58.7% to 84.3%.

In the experimental group (Table 7), consisting of participants with psychoses, no significant associations were recorded between the two factors (1 and 2); however, they both show a positive correlation with the total IPPML score.

The control group, consisting of participants diagnosed with schizophrenia (Table 8), shows positive associations between Factor 1 and Factor 2 (r = 0.739; *p* < 0.01), as well as with the total IPPML score (r = 0.951; *p* < 0.01). This finding suggests that the IPPML may statistically and practically predict dangerousness. Furthermore, the Psychiatric Forensic Dangerousness Index could be hypothetically applied in forensic medical decisions regarding the risk of committing antisocial and potentially harmful acts.

The total sample (*N =* 261) presents positive associations between Factor 1 and Factor 2 (r = 0.601; *p* < 0.01), as well as between Factor 1 and the total IPPML score (r = 0.959; *p* < 0.01). Additionally, Factor 2 is positively correlated with the total IPPML score (r = 0.804; *p* < 0.01), reinforcing the predictive value of the IPPML (Table 9).

### 3.3. Discriminant Validity

Discriminant validity, in this context, refers to the ability of the IPPML to effectively differentiate participants based on gender (male vs. female).

Comparisons presented in Table 10 indicate that Factor 1 does not show significant gender differences in the total sample (*N =* 261). However, Factor 2 (t = 9.054; *p* < 0.001) presents significant values in males, a trend that is also observed in the total IPPML score (t = 3.709; *p* < 0.001), where psychiatric dangerousness and medico-legal implications are higher.

A deeper analysis of gender differences in forensic psychiatric dangerousness was conducted based on participant group classification.

In the experimental group (*N =* 126), presented in Table 11, Factor 2, similar to the total sample, indicates a higher level of psychiatric dangerousness in males compared to females (t = 7.155; *p* < 0.001), consistent with the total IPPML score (t = 4.829; *p* < 0.001).

In the control group, Table 12 shows that Factor 1 presents higher means in males compared to females (t = 2.651; *p* < 0.01), likely due to the fact that all participants in this sample were diagnosed exclusively with schizophrenia.

For Factor 2 (t = 8.221; *p* < 0.001) and the total IPPML score (t = 5.095; *p* < 0.001), the results are consistent with those found in the total sample and experimental group. This indicates the instrument’s ability to differentiate based on gender, thus experimentally supporting the general medical perception of the dangerousness phenomenon.

These findings reinforce the discriminant validity of the IPPML, demonstrating that the tool is effective in differentiating psychiatric dangerousness based on gender.

In most criminal justice systems, the primary mission involves fulfilling two fundamental tasks: assessing the degree of responsibility of the offender and evaluating their future dangerousness and risk of recidivism. The ongoing debate regarding the introduction of scales in forensic psychiatric evaluation has mainly focused on the first task—namely, the relevance and effectiveness of scientific evidence in determining an offender’s responsibility.

In forensic psychiatry, assessing future dangerousness and predicting the risk of recidivism is gaining increasing importance and becoming a central issue in forensic evaluations. These assessments influence the therapeutic or security measures that must be implemented.

Modern criminal justice systems no longer emphasize punishment but rather focus on prevention through treatment.

To develop a scale for measuring dangerousness in forensic psychiatry, we followed a psychometric scale construction methodology and identified two relevant factors.

The reliability of the instrument ranges between 89.6% and 62.8% when administered to participants with psychoses who have undergone forensic psychiatric evaluations. When used exclusively for legally assessed patients, the reliability of the instrument decreases to 42.5%. On the other hand, when applied to participants with schizophrenia who have not undergone forensic psychiatric evaluation, reliability increases from 58.7% to 84.3%.

Regarding discriminant validity, Factor 1 does not indicate gender-based differences in the total sample. However, Factor 2 presents significant values among males, a pattern that is also reflected in the total IPPML score, where psychiatric dangerousness with forensic implications is notably higher.

## 4. Discussion

The primary objective of this study was to develop and validate the Dangerousness Index in Forensic Psychiatry (IPPML) to assess the risk of recidivism and dangerousness in forensic psychiatric evaluations.

Our study observed that the IPPML demonstrated adequate internal consistency and identified two main factors, Performance and Social, which explained a significant portion of the data variance. The identification of these two distinct factors aligns with previous findings that highlight the importance of both behavioral and social aspects in risk assessment [18].

Previous research has highlighted the importance of reliable tools in forensic psychiatry to assess the risk of recidivism and dangerousness, yet many existing tools lack comprehensive validation. Tools such as the HCR-20 and the VRAG have been used widely but have shown varying levels of predictive accuracy depending on the population and context [19,20]. In particular, our study highlights the need for tools that minimize examiner subjectivity. The Mekereș Psychosocial Internalization Scale, for example, addresses forensic bias by establishing clearer limits for aesthetic injury assessment, supplementing morphometric scales to evaluate the psychosocial impact of scarring [21]. Similarly, the Bee Cosmetic Surgery Scale (BCSS) assesses attitudes toward cosmetic surgery based on motivational factors, reflecting an intersection between bodily self-perception and social acceptance [22]. These examples underscore the necessity for nuanced assessment tools in forensic psychiatry, further justifying the role of the IPPML.

Assessing dangerousness and the risk of recidivism is crucial in forensic psychiatry as it directly influences therapeutic and security measures for individuals undergoing psychiatric evaluations. Studies have shown that structured risk assessment tools can aid in decision-making and potentially reduce recidivism rates [9].

Compared to existing assessment tools, the IPPML showed promising results with higher internal consistency and reliability across different participant groups. For example, while the V-RISK-10 demonstrated good predictive validity (AUC = 0.83) for inpatient violence [11], our findings suggest that the IPPML offers comparable or better reliability in forensic psychiatric evaluations. Moreover, the instrument’s reliability ranged from 89.6% to 62.8% among psychotic individuals undergoing forensic psychiatric evaluation, a range consistent with established tools like the HCR-20 [23].

Contrary to some existing studies, our findings indicated a higher level of psychiatric dangerousness with forensic implications in males. While previous research has suggested that gender differences in forensic psychiatric settings are minimal [24], our results support evidence that men may exhibit higher rates of violent tendencies and forensic psychiatric referrals [13].

The IPPML demonstrated strong internal consistency (α = 0.881) and reliability ranging from 89.6% to 62.8% in participants with psychoses undergoing forensic psychiatric evaluation. These findings align with previous research indicating that structured assessments generally provide more reliable results than unstructured clinical judgments [23].

This study’s findings underscore the potential of the IPPML as a robust tool for assessing dangerousness in forensic psychiatry, thereby aiding in medical decision-making. Given the variability in predictive validity of existing tools such as the Static-99 [25], our tool offers an alternative with strong reliability indicators.

The IPPML can be utilized by forensic psychiatrists to make more informed decisions regarding the risk of antisocial and potentially harmful acts in individuals undergoing psychiatric evaluations. Risk assessment tools have played a crucial role in judicial decision-making, influencing sentencing and security measures [26,27].

While the IPPML shows promise, caution should be exercised in its application, particularly in legally evaluated patients, where reliability was observed to decrease. Previous studies have highlighted similar challenges with other tools, such as the SAPROF, which did not perform as well in certain forensic settings [28].

The development of the IPPML represents a novel contribution to forensic psychiatry, providing a new, validated tool for assessing dangerousness and risk of recidivism. Given the emerging interest in AI-based risk assessments [29], this study provides a structured alternative grounded in traditional psychometric evaluation.

Although the Risk–Need–Responsivity (RNR) model has significantly advanced risk prediction and the tailoring of interventions, its practical implementation is challenged by difficulties in maintaining assessment accuracy, ensuring robust staff training, and adapting treatments to diverse environments—highlighting the need for continual refinement and strong organizational support [30]. In a related study, a cohort of 332 forensic patients discharged from highly secured Forensic Psychiatric Centers/Clinics in the Netherlands between 2004 and 2008 was followed, and latent class analysis (LCA) was performed to cluster patients based on psychopathology and criminal offenses. The predictive validity of the HKT-R clinical items was then assessed for each class using official reconviction data two and five years after discharge. These results underscore that varying risk and protective factors may influence different subgroups of patients, emphasizing the importance of considering patient heterogeneity in both risk assessment and intervention planning [31]. Forensic risk assessment tools, predominantly developed on Western samples, may not account for cultural differences; cautioned against using instruments like the PCL-R, VRAG, and Static-99 with Indigenous offenders due to cultural bias and limited cross-cultural validity [32].

Differences between urban and rural settings—such as firearm availability, social isolation, and access to mental health services—can lead to misestimation of risk when generic assessments are applied uniformly across these contexts [33,34,35,36,37].

Studies using homogeneous samples (e.g., only male patients with schizophrenia) risk masking subgroup differences and limiting generalizability, as demonstrated by validation studies of the HCR-20 that failed to capture sex-based variations [38].

To enhance the accuracy and generalizability of forensic risk assessments, future research should include more diverse samples and conduct subgroup analyses to explore how cultural, environmental, and demographic factors influence risk profiles [39].

A limitation of this study is the relatively small sample size and the need for further validation in diverse populations to ensure the generalizability of the IPPML. Similar concerns have been raised in studies validating the Forensic Inpatient Quality of Life questionnaire [40].

## 5. Limitations

The study presents several limitations that should be acknowledged. One limitation is related to the sample size and its generalizability. Although the study included 261 participants, a larger and more diverse sample is needed to enhance the applicability of the findings. The study population was limited to individuals undergoing forensic psychiatric evaluation and those diagnosed with schizophrenia, which may not fully represent all forensic psychiatric cases.

Another limitation is the lack of a longitudinal follow-up to assess the predictive validity of the IPPML in determining actual recidivism or violent behavior over time. Without long-term tracking of patient outcomes, the extent to which the tool effectively predicts future forensic risk remains uncertain. Future studies should evaluate the tool’s effectiveness in real-world forensic settings over extended periods.

The legal and cultural context in which the study was conducted may also limit the generalizability of the findings. Forensic psychiatric practices vary across jurisdictions, and the applicability of the IPPML in different legal and cultural settings remains to be explored. Further research should consider cross-cultural validation to ensure the tool’s effectiveness in diverse forensic populations.

The study also revealed variations in reliability across different subgroups. While the IPPML showed strong reliability in individuals undergoing forensic psychiatric evaluations, its reliability was lower among legally evaluated patients. This suggests that the tool’s effectiveness may differ depending on the forensic population, requiring additional refinement to improve its applicability.

Another concern is the potential for response bias, as the IPPML relies on self-reported responses. Participants may have underreported or misrepresented certain behaviors due to social desirability bias, which could affect the accuracy of the assessments. Future research should explore complementary assessment methods, such as structured clinical interviews or collateral information from medical records, to improve the validity of the tool.

Additionally, the study did not incorporate neurobiological or psychophysiological data, which have been increasingly explored in forensic psychiatric risk assessments. Research suggests that neuroimaging and psychophysiological markers, such as brain scans or hormonal indicators, could enhance the accuracy of risk prediction. The integration of such measures could improve the predictive capacity of the IPPML and provide a more comprehensive evaluation of forensic psychiatric risk.

To address these limitations, future studies should focus on validating the IPPML in larger and more diverse forensic psychiatric samples, conducting longitudinal studies to assess its predictive validity, and comparing its performance against existing risk assessment tools. Further research should also explore the integration of objective biological and behavioral indicators to enhance the accuracy of risk assessments. Despite these limitations, the IPPML remains a promising tool for forensic psychiatric evaluations, and addressing these challenges will strengthen its reliability and applicability in forensic setting.

## 6. Conclusions

The IPPML represents a promising tool for evaluating dangerousness and forensic psychiatric risk, combining empirical reliability with structured clinical judgment. By integrating psychometric rigor with forensic applicability, the IPPML has the potential to improve risk assessment, therapeutic interventions, and judicial decision-making in forensic psychiatry. Further validation is warranted to solidify its role as a standardized instrument for psychiatric risk evaluation.

## Figures and Tables

**Table 2:** Distribution and percentages of the experimental and control groups.

Variable	Category	Experimental Group (*N =* 126)	%	Control Group (*N =* 135)	%
Gender	Men	76	60.3	81	60.0
	Women	50	39.7	54	40.0
Educational Status	Middle School	36	28.6	8	5.9
	Vocational School	28	22.2	29	21.5
	High School	53	42.1	79	58.9
	Higher Education	9	7.1	19	14.1
Marital Status	Unmarried	87	69.0	44	32.6
	Married	11	8.7	73	54.1
	Divorced	21	16.7	11	8.1
	Cohabitation	1	0.8	6	4.4
	Widowed	6	4.8	1	0.7
Socio-Economic Level	Low	72	57.1	11	8.1
	Medium	52	41.3	76	56.3
	High	2	1.6	48	35.6
Place of Origin	Rural	69	54.8	53	39.3
	Urban	57	45.2	82	60.7

**Table 3:** IPPML items, means, and standard deviations by group.

Item	Experimental Group Mean (SD) (*N =* 126)	Control Group Mean (SD) (*N =* 135)	Total Mean (SD)	*p*-Value	Cohen’s d
IPPML 1	4.9127 (0.43854)	4.8370 (0.58854)	4.8736 (0.52193)	0.243	0.1451
IPPML 2	2.5635 (0.75362)	2.0444 (0.73166)	2.2950 (0.78516)	<0.001	0.6992
IPPML 3	1.1667 (0.50200)	1.1111 (0.43480)	1.1379 (0.46836)	0.339	0.1186
IPPML 4	2.8016 (1.24592)	1.6741 (1.18340)	2.2184 (1.33669)	<0.001	0.9288
IPPML 5	4.8413 (0.57149)	2.0963 (0.89671)	3.4215 (1.56849)	<0.001	3.6242
IPPML 6	2.9762 (1.07677)	1.5111 (1.15814)	2.2184 (1.33669)	<0.001	1.3086
IPPML 7	1.8333 (0.87407)	1.4296 (0.82445)	1.6245 (0.87090)	<0.001	0.4756
IPPML 8	2.0476 (1.05722)	1.6889 (0.92613)	1.8621 (1.00582)	0.004	0.3618
IPPML 9	2.2381 (1.01531)	2.0000 (0.88098)	2.1149 (0.95384)	0.044	0.2511
IPPML 10	3.2698 (1.03856)	2.1407 (1.11411)	2.6858 (1.21566)	<0.001	1.0471
IPPML 11	1.3333 (0.82946)	1.3926 (0.92314)	1.3640 (0.87798)	0.587	−0.0674
IPPML 12	3.9127 (1.72983)	3.0296 (1.85658)	3.4559 (1.84677)	<0.001	0.4915
IPPML 13	1.6984 (1.43259)	3.3630 (1.56266)	2.5594 (1.71456)	<0.001	−1.1087
IPPML 14	3.6032 (0.80079)	1.9333 (0.98648)	2.7395 (1.22833)	<0.001	−1.852
IPPML 15	4.1984 (0.75916)	2.3481 (0.74632)	3.2414 (1.19259)	<0.001	−2.4587
IPPML 16	4.6111 (0.75865)	1.4667 (1.15125)	2.9847 (1.85425)	<0.001	−3.2035
IPPML 17	4.2857 (0.71394)	2.2222 (0.72962)	3.2184 (1.25966)	<0.001	−2.8577
IPPML 18	4.4841 (0.85542)	1.3704 (0.98300)	2.8736 (1.81111)	<0.001	−3.3712
IPPML 19	3.9365 (1.11532)	1.3556 (0.95782)	2.6015 (1.65548)	<0.001	−2.4893
IPPML 20	3.1905 (0.87374)	1.2593 (0.72238)	2.1916 (1.25334)	<0.001	−2.417

**Table 4:** IPPML component factors and explained variance.

Component	Initial Eigenvalues	Rotation		
	Total	% of Variance	Cumulative %	Total
1	9.111	45.553	45.553	9.111
2	3.883	19.417	64.970	3.883
3	1.328	6.638	71.609	
4	1.259	6.296	77.904	
5	0.754	3.772	81.676	
6	0.668	3.341	85.017	
7	0.597	2.986	88.003	
8	0.412	2.062	90.065	
9	0.366	1.828	91.894	
10	0.318	1.590	93.484	
11	0.241	1.205	94.689	
12	0.207	1.036	95.725	
...	...	...	...	...
20	0.031	0.154	100.000	

**Table 5:** Factorial structure matrix using the varimax method.

Items	Factor 1	Factor 2	Communality
IPPML16	0.954	0.125	0.372
IPPML18	0.938	0.126	0.269
IPPML17	0.928	0.030	0.006
IPPML15	0.914	0.101	0.684
IPPML5	0.913	−0.108	0.844
IPPML19	0.883	0.049	0.813
IPPML20	0.856	0.107	0.813
IPPML14	0.823	0.294	0.788
IPPML13	−0.621	−0.019	0.733
IPPML10	0.585	0.365	0.475

**Table 6:** Reliability of the Dangerousness Index in Forensic Psychiatry (IPPML).

Scale	Experimental Group (*N =* 126)	Control Group (*N =* 135)	Total Participants (*N =* 261)
	α Cronbach	α Cronbach	α Cronbach
Factor 1	0.622	0.817	0.896
Factor 2	0.479	0.587	0.628
Total IPPML	0.425	0.843	0.881

**Table 7:** Correlation matrix of IPPML scales in the experimental group.

Dimension	Factor 1	Factor 2	Total IPPML
Factor 1	-		
Factor 2	−0.157	-	
Total IPPML	0.663 **	0.635 **	-

** Highly statistically significant if *p* < 0.01.

**Table 8:** Correlation matrix of IPPML scales in the control group.

Dimension	Factor 1	Factor 2	Total IPPML
Factor 1	-		
Factor 2	0.739 **	-	
Total IPPML	0.951 **	0.911 **	-

** Highly statistically significant if *p* < 0.01.

**Table 9:** Correlation matrix of IPPML scales in the total sample (*N =* 261).

Dimension	Factor 1	Factor 2	Total IPPML
Factor 1	-		
Factor 2	0.601 **	-	
Total IPPML	0.959 **	0.804 **	-

** Highly statistically significant if *p* < 0.01.

**Table 10:** Gender-based comparisons for IPPML (*N =* 261).

Variable	Gender	*N*	M	SD	t	df	*p*
Factor 1	Male	157	29.1019	10.77460	1.079	259	0.281
	Female	104	27.6346	10.71616			
Factor 2	Male	157	25.2166	4.93200	9.054	259	0.001
	Female	104	20.0673	3.74753			
Total IPPML	Male	157	54.3185	14.22892	3.709	259	0.001
	Female	104	47.7019	13.92691			

**Table 11:** Gender-based comparisons for IPPML (*N =* 126)—experimental group.

Variable	Gender	*N*	M	SD	t	df	*p*
Factor 1	Male	76	38.0658	4.78703	−0.167	124	0.868
	Female	50	38.2000	3.75798			
Factor 2	Male	76	27.6447	4.13990	7.155	124	0.001
	Female	50	22.9600	2.54719			
Total IPPML	Male	76	65.7105	5.07757	4.829	124	0.001
	Female	50	61.1600	5.31981			

**Table 12:** Gender-based comparisons for IPPML (*N =* 135)—control group.

Variable	Gender	*N*	M	SD	t	df	*p*
Factor 1	Male	81	20.6914	7.54593	2.651	133	0.01
	Female	54	17.8519	2.70155			
Factor 2	Male	81	22.9383	4.52865	8.221	133	0.001
	Female	54	17.3889	2.46803			
Total IPPML	Male	81	43.6296	11.47437	5.095	133	0.001
	Female	54	35.2407	4.65786			

## Data Availability

The data supporting this study’s findings are available upon reasonable request from the corresponding author.
